# Epidemiology of Orofacial clefts in Africa: Methodological challenges in ascertainment

**DOI:** 10.4314/pamj.v2i1.51705

**Published:** 2009-04-30

**Authors:** A Butali, P.A Mossey

**Affiliations:** 1 PhD candidate; 2 Professor of Craniofacial abnormalities, Department of Dental Public health and Health Psychology, Dundee Dental Hospital and School, Park Place, Dundee., Scotland U.K, DD1 4HR., (WHO collaboration centres for craniofacial abnormalities

## Abstract

**Background:**

To carry out a systematic review of the birth prevalence of cleft lip with or without cleft palate (CLP) and cleft palate (CP) in Africa based on available published data.

**Method:**

Using the Cochrane search strategy and the following keywords words “cleft palate”, “prevalence”, “incidence”, “cleft lip” and “Africa” to screen Ovid Medline {1966 to March 2007), Cinahl {1982-March 2007}, Pub Med, Scopus, and Web-Google. All identified published, prospective and retrospective studies on the birth prevalence of CLP and CP in Africa were included. The dates, location, sources, number of births (live births, still births, number of cleft cases, prevalence rates, sex ratio, cleft types, and clefts with associated anomalies were extracted.

**Results:**

Ascertainment of cases was through the hospitals. Overall there were 57 CL/P, 56 CL and 36 CP reported from all the studies. From seven studies combined, 21 males and 20 females had CL, 10 males and 22 females with CP and 26 males and 24 females with CL/P. There were 3 cases with CL/P, 2 with CP and 2 with CL from the three studies that reported clefts with associated anomalies.

**Conclusion:**

For an improved ascertainment of cleft cases, there is a need to set up a birth defects surveillance system in the form of a national birth registry. Future studies should then aim to include the entire population in geographically defined regions. Reliable data on incidence is an essential pre-requisite for studies into aetiology and prevention.

## Background

Orofacial clefts (OFC), comprising cleft lip with or without palate (CLP) and cleft palate (CP) are amongst the most common birth defects of the head and neck. Although, the overall prevalence varies between 1/600–1/700, this rate varies based on geographic and ethnic distribution [1].

Africa, the second largest continent in the world, has a population of 967 million people as of mid-2008 with about 400 million (42 percent) below age 15. With an annual growth rate of about 2.4 percent (2.8 in middle Africa and 0.8 in Southern Africa), the continent is projected to reach 1.9 billion people by 2050 [2].

Reports of birth prevalence of orofacial clefts from different African populations vary widely, from as low as 0.3/1,000 reported in Nigeria [3] to 1.65/1,000 reported in Kenya [4]. Determining the exact prevalence of orofacial clefts in Africa is important for public health reasons as the prevalence rates will help identify cluster areas and possible etiological factors, which will in turn help governments plan strategies for preventive measures and treatment.

The aim of this study is to retrospectively evaluate the evidence available on the birth prevalence of orofacial cleft in Africa and implications for future research. The specific objective is to carry out a systematic review of the birth prevalence of cleft lip with or without cleft palate (CLP) and cleft palate (CP) in Africa, based on available published data.

## Method

### Data Sources

A systematic literature search was conducted using electronic databases Ovid MEDLINE (1966 to 2007), CINAHL {1982-March 2007}, Cochrane Database. Using the Cochrane search strategy, studies were found by using the following keywords: “cleft palate,” “prevalence,” “incidence,” “cleft lip,” and “Africa.” In addition, a combination of the following terms was also used: “incidence and cleft lip,” “incidence and cleft palate,” “cleft lip and Africa,” “cleft palate and Africa,” and “incidence of cleft lip and palate and Africa”

To identify eligible papers, we searched through the references of papers that mention orofacial clefts in Africa and read published articles on the topic (full text and abstracts), in English, Afrikkans, and Somali languages (translation was carried out using the language translation tool on the internet).

### The study selection

All identified published prospective and retrospective studies of birth prevalence of orofacial clefts in Africa were considered for inclusion in the review. The studies included reports on prevalence of orofacial clefts; prevalence of congenital birth defects including orofacial clefts; those that presented adequate information on the methodology of study; and studies on orofacial cleft cases that presented for treatment with a defined sample population.

We excluded the following: studies limited to clinical features and clefts pattern without a mention of the prevalence rate, reports on the aetiology, social impact or un-operated and adult clefts without a mention of prevalence rates of orofacial clefts; and studies that did not include data from which the prevalence rates can be calculated.

### Data extraction

The following were extracted from the studies: dates and location of studies, source of data, birth number / total number of patient seen and number of cleft patients, birth outcomes (live births, still births, and patients for treatment), sex ratio and types of cases, cases with associated malformations.

In many of the studies, the birth prevalence rate, sex ratio and cleft types, and clefts with associated anomalies were published in readily accessible format. In some studies outcomes were published in the form of percentages. In these cases, we calculated the prevalence rates.

The sex ratio, cleft types and those associated with anomalies were presented using the number of cleft cases. All the studies stated criteria for diagnosis of orofacial clefts, clefts types, and associated anomalies.

The data obtained did not meet the requirements for a meta-analysis and thus we carried out a systematic literature review.

## Results

The search combination in the databases produced 131 hits in Ovid Medline (1966 to March 2007) (http://ovidsp.tx.ovid.com/spa/ovidweb.cgi); 33 hits in Pub Med (http://www.ncbi.nlm.nih.gov/pubmed), 3 hits in Scopus (http://www.scopus.com/scopus/home.url); Web-Google search gave 388 hits and no hits in CINAHL {1982-March 2007}.

Web – Google is a non-scientific search tool. It looks for a combination of two or more of the entered terms and even when these are not in context, they appear as hits. This explains the large number of hits using the above combination. Most of the hits were not relevant to the search criteria.

Further search through the computer databases (Ovid Medline, Pub Med, and Scopus), produced a combination of 33 hits which refer to orofacial clefts in Africa.

A thorough evaluation of these papers using the inclusion and exclusion criteria led to the exclusion of 27 papers leaving 7 papers that met the inclusion criteria.

The hand search produced 5 additional papers and a total of 11 papers (all published in peer-reviewed journals) were thus available for review.

Outcomes were OFC in infants, specifically cleft lip with or without cleft palate (CLP) and cleft palate only (CP). Isolated clefts without any congenital defects and orofacial clefts with associated anomalies were included in three of the studies (Kromberg and Jenkins, 1982; Krouf et al., 1986; and Sulieman et al., 2005) (Table 1).

**Table 1:** Study design, reported prevalence rates and sample sizes

Study	country	Years of data collection	D	Birth outcomes	Rates (95% CI)	Study design	Sample sizes			
							CL	CP	CL/P	All clefts
Simpkiss and Lowe 1961	Uganda	Dec 1956 to Sept 1957	2,068	LBs	1.45 (0.76-2.14)	RHB study	2		1	3
Khan 1965	Kenya	1 Nov 1963 to 30 Apr 1964	3,016	LBs	1.65 (1.08-2.22)	RHB study	3		2	5
Gupta B 1969	Nigeria	1 Feb and 31 of Jul, 1964	4,066	LBs	0.95 (0.46-1.44)	PHB study	1(1^m^)	1(1^f^)	2(1^m^) (1^f^)	4
Robinson D.C, Shepherd J.J 1970	Uganda	1968	67,143*	LBs	0.75 (0.62-0.88)	PHB study	19(9^m^) (10^f^)	6(2^m^) (4^f^)	22(12^m^) (10^f^)	47
Iregbulem 1982	Nigeria	1976 - 1980	21,624	LBs	0.30 (0.12-0.48)	PHB study	4 (2^m^) (2^m^)	1(1^m^)	3(2^m^) (1^m^)	8
Jennifer G.R Kromberg, T. Jenkins 1982	South Africa	Jan 1976 to Dec 1977	29,633	LBSBs	0.30 (0.13-0.47)	RHB study	1 (1) (1^f^)	3(3m )	5 (1)(2^m^) (2^f^) (1^uk^)	9a
Morrison G, Cronje A.S, Van Vuuren. I, OP’T Hof J 1985	South Africa	Jan 1983 to Jan 1984	9,377	LBs	0.33 (0.25-0.41)	RHB study	3	0	0	3
Khrouf N, 1986	Tunisia	4 Oct 1983 to 16 Jul, 1984	10,000	LBSBs	1.50 (1.18-1.82)	PHB study	6 (1)	5(2)	4 (1)	15 a
Ogle 1993	Zaire	Oct 1977 to Jun 1979	56,637	LBs	0.46 (0.32-0.60)	RHB study	13(8^m^) (5^f^)	1 (1^f^)	12)(4^m^) (8^m^)	26
Msamati et al 2000	Malawi	Jan 1998 and Dec 1999	25,562	LBs	0.67 (0.65-0.69)	RHB study	1(1^f^)	16(4^m^) (12^f^)		17
Sulaiman et al.2005	Sudan	1997 to 2000	15,890	LBs	0.90 (0.64-1.16)	RHB study	2(1^m^) (1^f^)	4 (4^f^)	7 (1)(2^m^) (5^f^)	13

* Estimated size of population served by the hospital. D: Denominator

a clefts with associated anomalies

( ) number of associated anomalies

m males

f females

LBs: live births, LBSBs: Live births and still births, RHB: Retrospective hospital based, PHB: Prospective Hospital based

For most of the studies, the birth prevalence rate was expressed by dividing the number of cleft cases born (numerator) by the population at risk (denominator) multiplied by 1,000 during a specified time period. The population at risk in most of the studies were live born infants in the hospitals and cases that presented for treatment within the study period. Two of the studies included still-births (Kromberg and Jenkins, 1982; Khrouf et al., 1986) in the population at risk and included still- born infants with clefts among the reported cases (Table 1).

Overall there were 58 CL/P, 55 CL and 37 CP reported from all the studies (isolated clefts and clefts associated with anomalies). From seven studies combined, 21 males and 20 females had CL, 10 males and 22 females had CP and 23 males and 27 females had CL/P. There were three cases with CL/P, two with CP and two with CL from the three studies that reported clefts with associated anomalies (Table 1).

## Discussion

### Prevalence

The reviewed studies on the birth prevalence of orofacial clefts in Africa suggest that the prevalence is low compared to Europe, America, and Asia. However, compared to population-based studies, these studies are small and hospital-based (Table 1), carry a high tendency for under ascertainment of cases, and are more liable to bias. Ascertainment may be difficult in countries where a high proportion of births may occur in areas remote from structured healthcare systems, meaning that records may be incomplete. This factor may have contributed to lower estimates from some countries. Although hospital-based estimates give an indication of rate, they are subject to biases making them not directly comparable with those where complete ascertainment is achieved.

In some studies, less than 30% of births take place in a hospital [5] and in most cases a large proportion of births occur at home [6]. In severe cases of congenital abnormalities such as hydrocephalus where obstetrical problems may arise, there is likely to be bias towards these cases and numbers may appear higher than is the true incidence. To address these problems, a prospective study is required, based on a captive population where every birth (within and outside the hospital) is recorded by observers who are specifically examining for these conditions [7].

It has been suggested that a high infant mortality rate, including unreported infanticide in children with lip or palatal clefts could account for the low incidence and birth prevalence rates [3, 8]. The extremely low incidence of consanguineous marriages among Nigerians has been reported as a possible reason for low prevalence rate of cleft lip and palate in Nigeria [3]. (In Nigeria, it is virtually impossible to marry a cousin no matter how far removed and as a consequence, the possibility of summation of any genetic trait is extremely remote.)

Some studies reported high rates for cleft lip and palate in Africa [4, 6, 9–11] compared to other studies that reported low rates [3, 12] (Table 2). Although some of the studies with reported high rates included live birth and / or still births in the base population, the observed population was small with only one source of ascertainment limiting the source of information (Table 2). The studies were hospital-based which not a true representation of the general population. The study from Tunisia had a mixture of North African and sub-Saharan African populations which may account for a high rate reported from the study [11].

**Table 2:** Epidemiology of orofacial clefts: Study characteristics

**Study**	**Study characteristics**
Simpkiss and Lowe 1961	In the study at Mulago hospital in Uganda stillborn were seen and it was not possible to do autopsies. It was also difficult to see all the stillborn babies because relatives took the body away very soon after delivery. Of these still born, 40 were not seen by them, but they relied on written reports in notes. Major abnormalities were recorded by the midwife or medical student delivering the mothers.
Khan 1965	The study observed congenital malformations on newborn infants in the first 24-48 hours, and only major abnormalities were recorded newborn were examined at Pumwani African Maternity hospital in Nairobi. In the large majority of patients there is no antenatal care or regular attendance at the clinics, either due to ignorance or lack of interest. A large number of deliveries also take place in women who come to the hospital from outlying areas.
Gupta 1969	All live births were examined within 24 hours of birth and findings were recorded on a standard proforma at Adeoyo Hospital in Ibadan, Nigeria. Where doubts were raised about the presence of congenital malformation, cases were re-examined at the infant welfare and B.C.G clinics. Autopsies were performed on the majority of infants who died during the neonatal period and on all the still births in this series. The following information was recorded on all mothers- age, parity, duration of pregnancy, complications of pregnancy, and attendance in antenatal clinics, booked and unbooked deliveries. Because of the lack of any standard education of the majority of mothers and the general unreliability of histories, it was not feasible to attempt a detailed investigation of the maternity history. Most of the mothers came from low socio-economic groups. The study included only congenital malformations which were apparent on macroscopically examination, clinically or at autopsy. Macroscopically and biochemical abnormalities, except where there were gross pathological changes were not included. When multiple malformations were encountered, the case was classified only under the major malformation, e.g. - Meningo-myelocele with talepis as meninigo-myelocele.
Robinson D.C, Shepherd J.J 1970	At each clinic, the child was examined by a surgeon, a paediatrician, a dental surgeon and a maternal and child health worker. Detailed history was recorded and a full examination made
Iregbulem 1982	Live births were examined for cleft and the palate including sub mucous clefting at the University of Nigeria Teaching Hospital Enugu. In addition, information was obtained on 360 clinical patients regarding their date of birth, sex, types of clefts, parental age, villages of origin, and the presence of other congenital malformations. Mothers were asked about previous abortions and about their antenatal history, including illnesses and drugs taken in first trimester of pregnancy. Full information was not obtained in every instance. Iregbulem reported a total of 65 associated congenital malformations in the 360 clinical cases
Jennifer G.R Kromberg, T. Jenkins 1982	Babies born before admission of the mothers to the hospital were excluded. Information was obtained from three sources: {i] the register of births in the nursery ward; {ii} the paediatric ward registers; and {iii} the mortuary records. From each of the studies ,details of stillbirths, neonatal deaths, congenital defects and multiple births were recorded and in each case the mother’s name and hospital number, date of admission and the outcome of the pregnancy {sex of baby and diagnosis} were noted. The files of all those infants reported as having congenital defects but in whom the diagnosis was in some doubt, as well as on those who were stillborn or died in the neonatal periods, were then drawn and carefully scrutinized for clarification or confirmation of diagnosis.
Morrison G, Cronje A.S, Van Vuuren. I, OP’T Hof J 1985	All the doctors in the area were alerted on the project through the newsletter of Cape Western Branch of the Medical Association of South Africa. Also notified were hospital in Zynberg, Simonstown, Kuilsrivier and Greenwood. The area is relatively sparsely populated and no problems were encountered in deciding whether or not to include any particular patient in the study group. The population and birth figures used in calculating the prevalence rates were obtained from the Central statistical Services used.
Khrouf N, 1986	Malformations were recorded as observed during the first 24 hours in 10000 infants prospectively on a day to day schedule. The population studied were of North African extraction, almost all urban. A majority of the women are white, many are racially mixed, very few are black. Cousin marriages are common (about 36% of the couples) and are mostly first cousin marriages. About 30% of the mothers are primiparas and majority do not work outside their home. Alcohol, drug and cigarette usage is almost negligible. Most women did not consult prenatally and many show up late in labour, and after a vaginal delivery they return home with their newborn after 24hours of observation. The socioeconomic background ranges from middle class over lower class to a number of women living under very basic conditions. In Tunis with surroundings, rate of hospital to home deliveries is 64 to 100
Ogle 1993	A total of 56,637 live births {the figure excludes 9 newborns: 4 non –Zairians, and 5 with syndromes} were examined for cleft in Mama Yemo Hospital. The 9 newborns were excluded because the study was aimed at the prevalence of cleft lip and palate amongst Zairians with non-syndromic clefts. Weekly records of births were reviewed and cases of clefts were noted.
Msamati et al 2000	Delivery and nursery records from the department of Obstetrics and Gynaecology and Paediatrics at Queen Elizabeth Central Hospital {QECH} were compiled. The QECH is a referral and University teaching hospital serving regions, which has 12 districts and a number of mission hospitals. As such, its patients are more representative of the population in southern Malawi. There were more affected females than males (with a ratio of 4:1 p<0.05)
Sulaiman et al 2005	The records of new-borns delivered in the four year period were collected from three hospitals in Khartoum :(1) Omdurman Maternity hospital; (2) Al-Rahibat Maternity hospital; and (3) Khartoum North General hospital. The records of 8,236 of the 53,895 births which took place in Omdurman Maternity Hospital were complete, as were those for 297 of the 16,725 births which took place in Khartoum North Maternity hospital. At Al- Rahibat Maternity hospital, proper documentation with regards to presence of cleft lip and palate, type of cleft, presence of other anomalies, and sex of the child was found for 7,357 births out of 7,828 recorded for the 4-years study period. Other records were missing or incomplete. The number of birth at each hospital during the 4-years period was analysed with respect to presence of cleft lip and palate, types of cleft, presence of other anomalies and sex of the child.

The longer the period over which observation is made, the more confidence can be placed in the significance of change [1]. It is ideal to have a longitudinal study extending over 12 months to 5 years, however, difficult or impossible follow up are likely limitations in developing countries [4].

Studies from South Africa reported similar prevalence rates (0.3/1,000, 0.33/1,000) for the same geographical area [12–13]. In one study [12], data were obtained retrospectively from hospital records between 1976 and 1977. These data were from three different sources and details of still births, neonatal deaths, congenital defects, and multiple births were recorded. The other study [13] was prospective between 1983 and 1984; data were collected on all live cases and those that presented for treatment from hospitals and private practices in West Cape. Although the reported prevalence rates appear similar, the first study had better method of ascertainment and the reported prevalence was more reliable.

The report of more CL in males and more CP in females from the African studies are consistent with the literature [14]. The overall report of CL/P being higher in males in the African studies is also consistent with the literature [14]. The wide range in the confidence intervals in most of the studies (Table 1) suggests that the precision is low, which may be as a result of poor ascertainment of cases.

### Selection bias

The identification of subjects only in hospitals (Table 2) by all of the studies carried out on the epidemiology of orofacial clefts in Africa is a potential source of selection bias. Selection bias occurs when the subjects included are not likely to be representative of the source population as seen in the reviewed studies (Table 1). Another source of bias is the insufficient amount or quality of data reported by the authors. For example, studies by Simpkiss and Lowe, (1961) and Khan, (1965) did not provide data for cleft palate in the population. These are evidence of poor ascertainment. The selection bias and lack of homogeneity in the reviewed African studies meant a meta-analysis would be meaningless.

### International comparisons

In the United States, the reported low prevalence rates for black Americans [15] are comparable to reported rates among Nigerians [3] and black South Africans [12]. Another study reported rates for whites in the United States [16] which are comparable to reported rates for white South Africans [13]. It is interesting to compare rates of other ethnic groups to those of Africans while recognising the limitations.

The difference in prevalence among whites and blacks in different geographical location points to the fact that there are different genetic components. Aetiology is thought to involve genetic predisposition to orofacial clefts coupled with environmental factors. The low prevalence figure of 0.41/1,000 reported in a hospital-based study in India [17] is similar to rates reported in hospital-based studies in Africa. Both places are developing and there is probably under ascertainment of orofacial cleft cases. Caution must however be taken when comparing rates of other continents to Africa because of the different types of ascertainment, sample sizes and method of analysis used in those studies.

## Conclusion

Hospital based studies are known to be prone to bias and under ascertainment of cases is likely from the reported studies. For an improved ascertainment of cleft lip and palate cases in sub-Saharan Africa, there is a need to set up a birth defects surveillance system. This was a recommendation of the WHO [1] in 2003, and having identified the current unsatisfactory situation, we recommend beginning with a hospital-based birth defects surveillance system and then progressing to a regional population-based system. Future initiatives should then aim to include the entire population in geographically defined regions, country by country, ultimately aiming towards a national births and birth defects registry. In the short term, cleft lip and palate might serve as a sentinel birth defect for the sake of epidemiology and a good birth defect to study, simultaneously establishing the infrastructure for inter-centre collaboration and the capacity for participating in research into prevention of clefts and improving quality of care.

Additional research to determine birth prevalence and in due course incidence of orofacial clefts in Africa is needed. Reliable data on incidence is an essential pre-requisite for studies into aetiology and prevention.

## Competing interests

None declared. This article was presented at the CLEFTSiS education meeting on the 17th of August 2006 at Perth Royal Infirmary in Perth Scotland U.K.

## Authors’ contributions

**Professor P A Mossey** initiated the project and designed the study; **Dr A Butali** carried out the systematic review and both authors contributed to the manuscript preparation.

## Acknowledgments

We acknowledge the assistance of Professor D R Stirrups who was one of the independent assessors of eligible studies.
